# Myasthenia gravis AChR antibodies inhibit function of rapsyn-clustered AChRs

**DOI:** 10.1136/jnnp-2019-322640

**Published:** 2020-03-12

**Authors:** Hakan Cetin, Richard Webster, Wei Wei Liu, Akiko Nagaishi, Inga Koneczny, Fritz Zimprich, Susan Maxwell, Judith Cossins, David Beeson, Angela Vincent

**Affiliations:** 1 Nuffield Department of Clinical Neurosciences, University of Oxford, Oxford, Oxfordshire, UK; 2 Department of Neurology, Medical University of Vienna, Vienna, Austria; 3 Institute of Neurology, Medical University of Vienna, Vienna, Austria

## Abstract

**Objective:**

Direct inhibition of acetylcholine receptor (AChR) function by autoantibodies (Abs) is considered a rare pathogenic mechanism in myasthenia gravis (MG), but is usually studied on AChRs expressed in cell lines, rather than tightly clustered by the intracellular scaffolding protein, rapsyn, as at the intact neuromuscular junction. We hypothesised that clustered AChRs would provide a better target for investigating the functional effects of AChR-Abs.

**Methods:**

Acetylcholine-induced currents were measured using whole-cell patch clamping and a fast perfusion system to assess fast (<2 min) functional effects of the serum samples. The sensitivity, specificity and rapidity of the system were first demonstrated by applying maternal AChR-Ab positive plasmas known to inhibit fetal AChR function in TE671 cells. Eleven previously untested AChR-Ab positive MG sera, 10 AChR-Ab negative MG sera and 5 healthy control sera were then applied to unclustered and rapsyn-clustered human adult AChRs in CN21 cells.

**Results:**

The maternal AChR-Ab positive plasmas reduced fetal AChR currents, but not adult AChR currents, by >80% within 100 s. Only 2/11 AChR-Ab positive sera inhibited AChR currents in unclustered AChRs, but 6/11 AChR-Ab positive sera compared with none of the 10 AChR-Ab negative sera (p=0.0020) inhibited rapsyn-clustered AChR currents, and current inhibition by the AChR-Ab positive sera was greater when the AChRs were clustered (p=0.0385). None of the sera had detectable effects on desensitisation or recovery from desensitisation.

**Conclusion:**

These results show that antibodies can inhibit AChR function rapidly and demonstrate the importance of clustering in exploring pathogenic disease mechanisms of MG Abs.

## Introduction

Myasthenia gravis (MG) is an autoimmune disorder of the neuromuscular junction leading to weakness and increased fatigability. Autoantibodies (Abs) directed against the acetylcholine receptor (AChR), usually of IgG1 or IgG3 subclass, can be detected in about 80% of cases by radioimmunoprecipitation assays (RIAs).^1 2^ The pathological mechanisms include complement-mediated damage of the postsynaptic membrane, increased AChR internalisation followed by degradation and, apparently rare, direct inhibition of AChR function.^3^ The inhibitory antibodies are assumed to interfere with the acetylcholine (ACh) binding site of the receptor.^4 5^

Using electrophysiological and ^22^Na^+^ influx studies to measure AChR function on cell lines, a variable proportion of AChR-Ab negative sera were also found to inhibit AChR function,^6 7^ and it was hypothesised that other circulating antibodies were involved. Some of the AChR-Ab negative sera were identified retrospectively to contain antibodies binding to muscle-specific kinase (MuSK)^8^ or to AChRs that were clustered with the intracellular protein rapsyn (ie, clustered AChR-Abs), as they are at the neuromuscular junction.^9^ Approximately 5%–10% of the remaining sera were negative for all tests, although a small number have LRP4 antibodies.^10^

We recently showed that clustered AChRs have different recovery kinetics from desensitisation, suggesting that their functional properties are modified by interaction with rapsyn.^11^ Here, we performed a comprehensive functional analysis of the effects of MG sera on human adult and fetal AChRs expressed with and without rapsyn-induced clustering.

## Methods

### Patients and samples

The MG samples were prospectively collected in the Department of Neurology, Medical University of Vienna, from patients diagnosed by clinical, electromyographic or serological criteria (table 1). All sera were frozen at –20°C until use. For positive controls, two plasmapheresis samples from previous studies of mothers whose fetuses developed arthrogryposis multiplex congenita (AMC2 and AMC6) in utero^12 13^ were retrieved from the Oxford –20°C archives. All samples were screened using RIAs for AChR-Abs and MuSK-Abs and those that were negative were then tested by cell-based assays (CBAs) for clustered AChR-Abs, MuSK-Abs and LRP4-Abs as used routinely by the Oxford group. Patients whose sera were negative on all tests (SNMG) were included only if the Tensilon test and/or the repetitive nerve stimulation was positive. All samples were heated to 56°C for 30 min to inactivate complement, centrifuged at 13 000g for 5 min at room temperature (RT), dialysed against extracellular solution (ES) (Slide-A-Lyzer MINI Dialysis Device, 20K MWCO, Thermo Fisher Scientific), filter-sterilised (Corning 0.2 µm syringe filters, Sigma-Aldrich) and diluted 1:20 in ES before use.

**Table 1:** Clinical data of 21 patients with myasthenia gravis

Sample no	Subgroup	Sex/age at onset, years	Disease duration, years*	Diagnostic testing	PIS/MGFA classification*	Therapies given* (thymus histology)	AChR RIA, nmol/L	AChR CBA	MuSK CBA	CN21 without rapsyn current inhibition, % (no of cells)	CN21 with rapsyn current inhibition, % (no of cells)
1	AChR	F/14	17	Decr + Tens +	IIIa	APR 5 mg	38.7	ND	ND	30.3±3.3† (3)	22.9±5.3† (4)
2	AChR	F/59	8	Decr + Tens +	IIIb	APR, AZA, THX (normal)	7.6	ND	ND	16.9±9.1 (3)	9.9±3.1 (3)
3	AChR	F/30	2	Decr + Tens +	I	APR, THX (hyperplasia)	>300	ND	ND	11.3±1.7 (3)	14.8±2.9 (3)
4	AChR	F/35	9	Decr – Tens +	MM	MMF, THX (thymoma)	15.6	ND	ND	10.0±3.3 (3)	22.4±8.8† (3)
5	AChR	M/58	2	Decr – Tens +	MM	APR, AZA	14.8	ND	ND	10.4±4.9 (3)	10.8±3.4 (3)
6	AChR	F/35	8	Decr + Tens +	I	APR, THX (hyperplasia)	11.8	ND	ND	16.7±5.8 (3)	6.4±4.0 (3)
7	AChR	M/72	1	Decr + Tens +	IIb	Naive	5.8	ND	ND	28.5±15.3† (3)	39.5±12.1† (4)
8	AChR	M/54	3	Decr + Tens +	PR	APR, AZA, THX (normal)	7.4	ND	ND	11.1±3.1 (3)	2.3±4.2 (3)
9	AChR	M/65	3	Decr – Tens +	PR	APR, THX (normal)	5.4	ND	ND	10.1±4.1 (3)	29.1±8.4† (3)
10	Clustered AChR	M/42	0.5	Decr + Tens +	PR	APR, AZA, PLEX	<0.25	Pos	Neg	14.5±7.5 (7)	24.0±1.2† (4)
11	Clustered AChR	M/48	20	Decr + Tens +	PR	AChEI, THX (normal)	<0.25	Pos	Neg	9.4±3.3 (3)	27.9±0.2† (3)
12	MuSK	F/22	6	Decr – Tens +	PR	AZA, THX (hyperplasia)		Neg	Pos	13.4±4.3 (3)	11.0±2.4 (3)
13	MuSK	F/38	4	Decr + Tens –	IIIb	APR, AZA, PLEX		Neg	Pos	9.1±2.1 (3)	8.7±1.4 (3)
14	MuSK	F/28	12	Decr + Tens –	IIIb	APR, AZA		Neg	Pos	18.2±3.4 (3)	10.6±3.1 (3)
15	MuSK	F/23	19	Decr – Tens –	IIb	APR, RTX, THX (normal)		Neg	Pos	13.1±1.2 (3)	7.4±1.0 (3)
16	MuSK	F/41	16	Decr + Tens +	PR	AZA 100, THX (normal)		Neg	Pos	0.4±4.7 (3)	11.3±2.6 (3)
17	SNMG	M/14	11	Decr + Tens +	PR	APR		Neg	Neg	10.9±1.4 (3)	11.8±2.4 (3)
18	SNMG	M/55	1	Decr – Tens +	IIb	Naive		Neg	Neg	19.1±3.7 (3)	1.8±11.9 (3)
19	SNMG	M/28	5	Decr + Tens –	IIb	APR, MMF, THX (hyperplasia)		Neg	Neg	16.6±3.1 (3)	14.6±1.9 (4)
20	SNMG	F/25	22	Decr + Tens –	IIIb	TAC, THX (hyperplasia)		Neg	Neg	14.4±2.8 (3)	10.1±1.1 (3)
21	SNMG	F/25	6	Decr – Tens +	IIa	AZA, THX (thymitis)		Neg	Neg	25.8±2.7† (3)	8.9±3.0 (3)

*At the time of blood sampling.

†Significant current inhibition higher than 3 SD from mean value of five HC sera.

AChEI, acetylcholinesterase inhibitor; AChR, acetylcholine receptor; APR, prednisolone; AZA, azathioprine; BSA, bovine serum albumin; CBA, cell-based assay; CSR, complete stable remission; Decr, decrement at repetitive stimulation; HC, Healthy control; MGFA, Myasthenia Gravis Foundation of America; MM, minimal manifestation; MMF, mycophenolate mofetil; MuSK, muscle-specific kinase; ND, not determined; ns, Not significant; PBS, Phosphate buffered saline; PIS, postintervention status; PR, pharmacological remission; RIA, radioimmunoprecipitation assay; RTX, rituximab; SNMG, seronegative myasthenia gravis; TAC, tacrolimus; Tens, Tensilon test; THX, thymectomy.

### Cell culture

TE671 cells express only the fetal AChR isoform and were used to test the AMC plasmas. CN21 cells derive from the TE671 cell line that was stably transfected with excess cDNA encoding the human AChR ε-subunit, so that the adult AChR isoform is the predominant form expressed.^14^ Both cell lines were cultured at 37°C and 5% CO_2_ in Dulbecco-modified essential medium(DMEM; Sigma-Aldrich) supplemented with 10% fetal calf serum and 1% antibiotics/antimycotics (ie, penicillin G, streptomycin and amphotericin B, PSA) (Invitrogen).

### Transfections

The cells were seeded at 2×10^5^ cells per well in 6-well plates. On the following day, cells were detached from the plates using trypsin/EDTA, centrifuged at 1200g for 4 min and resuspended in prewarmed growth medium without PSA. For transfection with rapsyn, a corresponding amount of the cell suspension containing 1.25×10^6^ cells was again centrifuged at 1200g for 5 min and resuspended in 250 µL resuspension buffer R containing 20 µg of DNA at a concentration of 1 µg/µL. Cells were electroporated with either pIRES2-EGFP (‘no rapsyn’) or pIRES2-RAPSN-EGFP (‘rapsyn’) at a voltage of 1100 V and a pulse width of 30 ms using the NEON transfection system (MPK5000, Invitrogen). Immediately after electroporation, cells were resuspended in prewarmed growth medium without PSA and plated onto poly-L-lysine-coated glass coverslips. The medium was replaced by growth medium with PSA the following day. CN21 cells were patched between 2 or 3 days after transfection.

### Fluorescence microscopy

To demonstrate AChR clustering, the cells were visualised on an Olympus IX71 fluorescence microscope. Between 2 or 3 days after transfection, cells were incubated with mAb C7 for 1 hour at RT, which binds the extracellular domain of the AChR δ-subunit,^15^ diluted 1:1000 in staining medium (DMEM containing 20 mM Hepes and 1% bovine serum albumin (BSA)), before being washed three times with staining medium. Cells were fixed with 3% paraformaldehyde at RT for 10 min, washed three times with phosphate-buffered saline (PBS) and incubated with secondary antibody Alexa Fluor 594 goat anti-mouse IgG (H+L) (catalogue no. A11005; Invitrogen; RRID: AB141372) diluted 1:750 in staining medium. Cells were washed three times in PBS and mounted in fluorescence mounting medium (Dako Cytomation, Glostrup, Denmark). Images were captured using Simple PCI (Digital Pixel).

### Electrophysiology

All experiments were performed at RT using the whole-cell patch clamp configuration. Recording pipettes were made of thin-walled borosilicate glass (GC150TF-10, Harvard Apparatus). Pipette tips were fire polished to a final resistance of ~3 MΩ (MF-900 Microforge, Narishige). The ES contained (in mM): NaCl 150, KCl 2.8, HEPES 10, MgCl_2_ 2, CaCl_2_ 2 and glucose 10 with pH adjusted to 7.4 using NaOH. The pipette solution contained (in mM): NaCl 4, KCl 144, HEPES 10, MgCl_2_ 2, ATP 2 and EGTA 10 with pH adjusted to 7.2 using KOH. All measurements were performed at a holding potential of −60 mV. Currents were amplified using an Axopatch-1D amplifier (Molecular Devices) and, after filtering at 5 kHz, sampled to hard disk at 25 kHz. Series resistance was compensated for at least 95% with residual holding potential errors <2 mV.

Fast solution exchange was accomplished using the modified HSSE-2/3 application system (ALA Scientific Instruments). A four-barrel perfusion pipette with a tip diameter of ~300 µm was used to switch between ES without ACh, ES with 1 mM ACh, diluted serum without ACh and diluted serum with 1 mM ACh. Application times were fast with 10%–90% current rise times <10 ms in the whole-cell configuration.

Preliminary experiments were performed to test the fast perfusion system and demonstrate acute inhibitory effects of the open-channel blocker fluoxetine, the irreversible competitive antagonist α-bungarotoxin (BTX) (10 µM and 50 nM, respectively) and the two AMC sera.

### ACh application protocol

**Figure 1:**
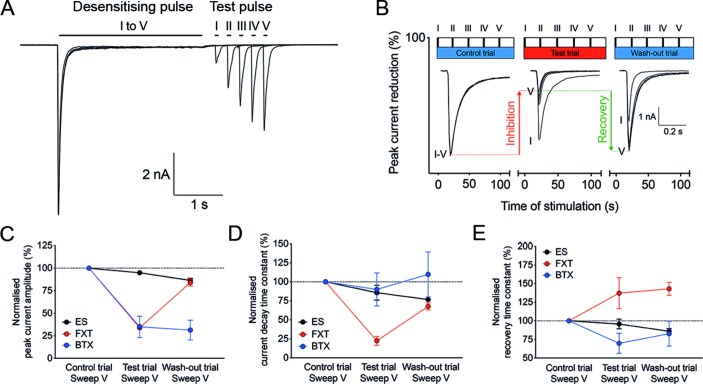
The acetylcholine (ACh) application protocol and the effect of fluoxetine (FXT) and bungarotoxin (BTX) on unclustered acetylcholine receptor whole-cell currents. (A) ACh application protocol. (B) The ACh application protocol was repeated three times on each cell according to three trials: a control trial (ie, perfusion of cells with extracellular solution (ES) only, left blue bar), a test trial (ie, perfusion of cells with FXT, BTX or serum diluted in ES, red bar) and a wash-out trial (ie, perfusion of cells again with ES only, right blue bar). The inlaid current traces display the desensitising pulses of the five sweeps in each trial and show current inhibition by FXT. (C) Both FXT and BTX extensively reduced peak current amplitudes within 100 s (66.1%±0.7% (n=3, p<0.0001) and 65.0%±11.8% (n=3, p<0.0001), respectively) compared with ES alone (5.1%±1.7% (n=8)). On washing out, only the effect of FXT was reversible. (D) As expected for an open-channel blocker, FXT produced a significantly shorter mean current decay time constant (–77.5%±5.9% (n=3) compared with –14.3%±9.7% in ES (n=8), p=0.0043; negative values correspond to a reduction of current decay time constants and thus facilitation of desensitisation). (E) FXT was also associated with prolonged recovery time constants (37.3%±20.9% in FXT (n=3), compared with –4.3%±6.5% in ES (n=8), p=0.0283). By contrast, the inhibition by BTX did not reverse on washing out, as expected for this irreversible inhibitory toxin (C). In addition, neither the current decay time constants (–10.0%±21.8%, n=3, ns) or the recovery time constants (–30.3%±13.5%, n=3, not significant) were different from ES (D and E).

The ACh application protocol is described in detail elsewhere,^11^ and was modified here for experiments on the functional effect of sera and known inhibitors. The protocol is illustrated in figure 1. It consisted of a series of five sweeps (I–V) with two consecutive 1 mM ACh pulses in each sweep (a ‘desensitising pulse’ of 3 s duration at the start of each sweep was followed by a ‘test pulse’ of 50 ms duration). The interval between both pulses increased by 250 ms with each sweep. The duration of each sweep was 25 s. Accordingly desensitisation pulses in sweeps I–V of the application protocol were applied at 0, 25, 50, 75 and 100 s.

In each cell, the ACh application protocol was repeated three times corresponding to three trials: (1) a ‘control trial’, in which the cell was perfused with ES only, (2) a ‘test trial’, in which the cell was perfused with serum diluted in ES and (3) a ‘wash-out trial’, in which the cell was again perfused with ES only. The duration of each trial consisting of five sweeps was 125 s. Analysis of desensitisation and recovery from desensitisation were assessed as described in our previous study.^11^

### Statistical analysis, randomisation and blinding

All data were analysed using GraphPad Prism V.7.0a. Clampfit V.10.5 was used for current decay time constant calculation. Randomisation was achieved by the order of experiments on different groups, that is, experiments were performed alternately on cells with and without rapsyn rather than testing each group in a block. Blinding was not feasible as all experiments and analyses were performed by HC. However, the effect of a potential bias was reduced by defining strict methodological inclusion criteria (ie, residual holding potential errors of <2 mV, current amplitudes of <20 nA and 10%–90% current rise times of <10 ms) and by the application of automated algorithms for data analyses as implemented in Clampfit V.10.5. Results are presented as mean±SEM (unless otherwise stated) and compared using the two-tailed unpaired t-test or Fisher’s exact test. One-way analysis of variance (ANOVA) was used for comparisons of more than two groups followed by Dunnett’s multiple comparison test. Linear regression analysis was performed to determine correlations between continuous variables. A two-sided p value<0.05 was considered significant.

## Results

### Testing AChR current amplitudes, desensitisation and recovery from desensitisation

The same application protocol was used for all samples (figure 1A). AChR current inhibition was defined by the reduction of current amplitudes during the test trial and calculated by the per cent ratio of the desensitisation pulse amplitudes between sweep V in the test trial and sweep V in the control trial (as shown for fluoxetine in figure 1B, C). The effect on desensitisation was defined by the per cent ratio of current decay time constants between sweep V of the test trial and sweep V of the control trial (figure 1D). The effect on AChR recovery from desensitisation was calculated as the per cent ratio of recovery time constants between the test trial and the control trial (figure 1E). The results clearly show fast irreversible inhibition by BTX (50 nM) and fast inhibition by fluoxetine (10 µM); only fluoxetine was reversible during the wash-out phase.

### Effects of fast perfusion of AMC sera on fetal AChR currents

**Figure 2:**
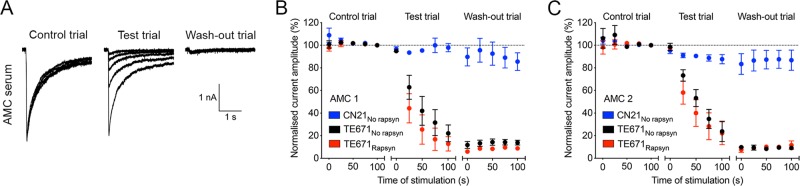
Time course of fetal acetylcholine receptor (AChR) current inhibition by arthrogryposis multiplex congenita (AMC) sera. Sample trace displaying the effect of AMC 1 on clustered fetal AChRs expressed in a TE671 cell (A). Desensitisation pulse amplitude reduction during the test trial was 88.7%. The time course of mean desensitisation pulse amplitudes in sweeps I–V of the control, test and wash-out trial is displayed for AMC plasma 1 (B) and 2 (C). Current inhibition during the test trial by both AMC sera was fast, extensive and irreversible during the wash-out trial. The effect of both AMC sera was not statistically different between unclustered and clustered fetal AChRs as expressed in TE671 cells (AMC 1: F_(1,20)_=3.7, p=0.0679, two-way analysis of variance (ANOVA); AMC 2: F_(1,20)_=1.9, p=0.1814, two-way ANOVA).

To test whether the, much larger, antibodies could inhibit as rapidly as fluoxetine and BTX, we first tested two plasmapheresis samples from mothers of AMC fetuses whose AChR-Abs were shown previously to inhibit currents in fetal AChR-expressing oocytes, or in fetal AChR expressing TE671 cells after 30 min incubation.^13^ Both maternal AMC samples inhibited AChR currents in TE671 cells, but not in the adult AChR predominant CN21 cells, within the 100 s time frame (figure 2A–C). As previously found, the inhibition of fetal AChR currents was irreversible and function was not restored during the wash-out phase (figure 2B, C).

### Patient characteristics

The MG cohort consisted of typical patients with MG presenting to the Department of Neurology in Vienna over a period of 10 years (table 1). The ages ranged from 14 to 72 years, and 57% were females. Disease duration at time of blood sampling was highly variable ranging from 0.5 to 22 years. Diagnosis was confirmed by AChR or MuSK RIA positivity, by neurophysiological evidence of neuromuscular junction defects, and/or by a positive response to Tensilon application. According to the Myasthenia Gravis Foundation of America (MGFA) classification and the postintervention status, nine patients had minimal manifestations (MM) or were in pharmacological remission (PR), two were MGFA I, and the remaining patients were classed as MGFA II to III, many with bulbar predominance. These characteristics are typical of patients presenting to a tertiary centre, many having been treated previously.

Based on in-house CBAs in routine diagnostic use, the sera were grouped in AChR-Ab positive sera (RIA positive (n=9) or RIA negative/clustered AChR positive (n=2)) and AChR-Ab negative sera consisting of MuSK-Ab positive sera (RIA/CBA-positive, n=5) or SNMG sera (CBA-negative for AChR-Abs, MuSK-Abs and LRP4-Abs, n=5).

### AChR current inhibition by AChR-Ab positive sera is facilitated by rapsyn

**Figure 3:**
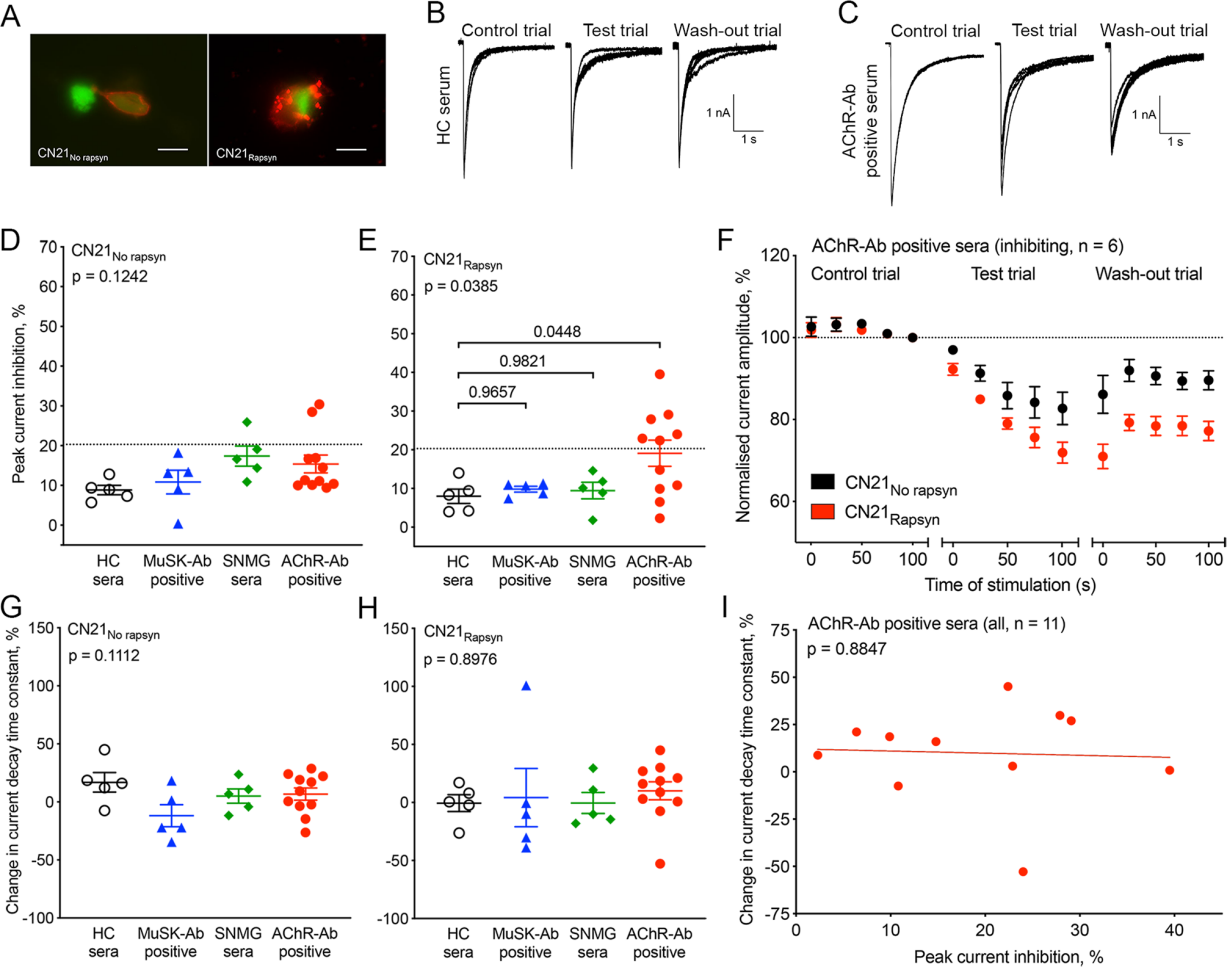
Acetylcholine receptor (AChR) whole-cell current inhibition by myasthenia gravis (MG) sera. (A) In CN21 cells, the cotransfection with rapsyn cDNA resulted in a change of AChR distribution (red staining, AF594) from a linear peripheral staining (left image) to receptor aggregation on the cell surface (right image). Scale bar=10 µm. Sample traces displaying the effect of one HC serum (B) and AChR-Ab positive serum 1 (C) on clustered adult AChRs expressed in a CN21 cell. Desensitisation pulse amplitude reduction during the test trial was 9.9% by the HC serum and 38.6% by AChR-Ab positive serum 1. (D) The difference of mean current inhibition between HC, muscle-specific kinase-Ab positive, SNMG and AChR-Ab positive sera was not significant in CN21 cells expressing unclustered adult AChRs (p=0.1242, one-way analysis of variance (ANOVA)). (E) In CN21 cells cotransfected with rapsyn to cluster adult AChRs, by contrast, current inhibition by AChR-Ab positive sera was significantly greater compared with the other MG sera (p=0.0385, one-way ANOVA followed by Dunnett’s multiple comparisons test to compare the various MG sera with HC sera). The dotted line in (D) and (E) represents the cut-off value to denote current inhibition (ie, mean inhibition by HC sera+3 SDs). (F) The time course of mean desensitisation pulse amplitudes in sweeps I–V of the control, test and wash-out trial is displayed for the AChR-Ab positive sera with significant current inhibition (n=6). Current inhibition by the six AChR-Ab positive sera was faster and stronger when the AChRs were clustered (F_(1,50)_=22.1, p<0.0001, two-way ANOVA). All desensitisation pulse amplitudes were normalised to the desensitisation pulse amplitude of sweep V in the control trial. In CN21 cells without rapsyn (G) and cotransfected with rapsyn (H), mean current decay time constant changes during the test trial were not significantly different in the MG sera as compared with HC sera (one-way ANOVA). (I) There was no correlation between mean current inhibition and mean current decay time constant changes during the test trial as shown for the 11 AChR-Ab positive sera tested on CN21 cells cotransfected with rapsyn (R^2^=0.002, p=0.8847, linear regression). Each serum was tested on at least three cells.

All sera were tested on CN21 cells expressing unclustered adult AChRs and on CN21 cells cotransfected with rapsyn to induce adult AChR clustering (figure 3A). Some sera inhibited the ACh-induced currents; inhibition was fast and could clearly be discerned during the test trials. As examples, figure 3B shows a sample trace from testing an HC serum and figure 3C a sample trace from an AChR-Ab positive serum.

The mean inhibition of whole-cell currents after 100 s exposure to HC sera was 8.0%±4.1% in clustered AChRs (mean±SD, n=5). For individual MG sera, any whole-cell current inhibition higher than 3 SDs from this mean (ie, 20.3%) was considered significantly increased. Unclustered AChR currents were inhibited by 2/11 AChR-Ab positive sera, by 0/5 MuSK-Ab positive sera and by 1/5 SNMG sera (table 1). The difference of mean current inhibition between the AChR-Ab positive and the other MG or HC sera was not significant (8.8%±1.2% in HC sera, 10.8%±3.0% in MuSK-Ab positive sera, 17.4%±2.5% in SNMG sera and 15.4%±2.2% in AChR-Ab positive sera, p=0.1242, one-way ANOVA; figure 3D). Rapsyn-clustered AChR currents, by contrast, were inhibited by 6/11 AChR-Ab positive sera and by 0/10 AChR-Ab negative MG sera (p=0.0020, Fisher’s exact test, table 1), and the mean current inhibition by AChR-Ab positive sera was significantly higher than the other MG or HC sera (8.0%±1.9% in HC sera, 9.8%±0.8% in MuSK-Ab positive sera, 9.4%±2.1% in SNMG sera and 19.1%±3.4% in AChR-Ab positive sera, p=0.0385, one-way ANOVA; figure 3E).

Although there were trends in both AChR-Ab positive and negative treated currents over time, the clustered AChR-Ab current amplitudes did not fully recover remaining different to those in HC sera (current reduction of 22.4%±2.3% by AChR-Ab positive sera at the end of the wash-out trial (figure 3F) compared with a current reduction of 8.4%±2.5% by HC sera, p=0.0024, unpaired t-test).

Four out of six patients with MG with sera characterised by clustered AChR current inhibition had clinically improved and were in PR or MM, and the remaining two patients with MG were still clinically active (MGFA II and III). Three out of six patients had undergone thymectomy (one patient diagnosed with thymoma) and four out of six patients were treated with immunosuppressive agents at the time of blood sampling. There was no correlation between AChR-Ab titres and clustered AChR current inhibition (table 1).

### Current inhibition is independent of desensitisation and irreversible

It was possible that facilitation of desensitisation or impaired recovery from desensitisation was responsible for the current inhibition by AChR-Ab positive sera. In this case, a correlation between the degree of inhibition and the current decay time constant change in individual cells would be expected. However, current decay time constant changes during the test trial, although variable, were not different between the MG and HC sera (figure 3G, H); moreover, for the AChR-Ab positive sera, there was no correlation between current inhibition and changes in current decay time constants (R^2^=0.002, p=0.8847, linear regression; figure 3I), nor between current inhibition and changes in recovery time constants (R^2^=0.002, p=0.8853, linear regression; data not shown).

## Discussion

AChR-Abs in MG bind variably to the main immunogenic regions and other epitopes on each of the two α-subunits and thus are able to cross link adjacent AChRs and cause internalisation and loss of the membrane receptors. In addition, the IgG1 or IgG3 AChR-Abs can activate complement leading to damage of the postsynaptic membrane. Both these mechanisms are time and temperature dependent. Direct inhibition of AChR function by the AChR-Abs has been studied in vitro, usually at RTs, using a variety of mouse, rat or human cell lines, transfected cells and occasionally intact neuromuscular junctions, but the results have been variable, perhaps because the conditions did not usually reflect the physiological situation at the neuromuscular junction where AChRs are packed as densely as 10 000 per µm^2^.^16^ Here, we hypothesised that testing the function of clustered AChRs would be the most appropriate method to detect inhibitory antibodies, and that any such effect would be irreversible. We found that 6/11 AChR-Ab positive sera (including the two that only bound to clustered AChRs) inhibited AChR currents of clustered adult AChRs in the CN21 cell line and that the inhibition did not wash out during the time of study. The results were as rapid, but not as marked, as those achieved by the AMC maternal sera inhibiting fetal AChR currents in TE671 cells.

There have been a number of reports that seropositive MG sera as well as monoclonal antibodies directed against the ACh binding site of the receptor (eg, the WF6 monoclonal antibody specifically binding to the α-subunit)^17^ can inhibit AChR function.^4 5 13 18 19^ Antibodies directed against other extracellular AChR epitopes (eg, the WF20 monoclonal antibody binding distant from the ACh binding site), by contrast, have not been associated with current inhibition.^4^ In one study on mouse myotubes, the effect mediated by IgG purified from four seropositive patients with MG proceeded to an irreversible block after 2 min incubation.^20^ This corresponds well to our data revealing irreversible current inhibition by AChR-Ab positive sera after 125 s incubation. In the previous study, however, it was hypothesised that the binding of specific antibodies to AChRs might proceed in several stages from low-affinity reversible inhibition to higher affinity irreversible inhibition of AChR function,^20^ which could be mediated by conformational changes of the AChR induced by the antibodies (such as desensitisation). We evaluated the effect of MG sera on desensitisation in human AChRs for the first time by the analysis of current decay time constants, and found no correlation between current inhibition and desensitisation, indicating that AChR current inhibition was not mediated by increased desensitisation. Although this conclusion is tentative given the variability between the responses, we feel that competitive antagonism is likely to be the underlying mechanism of AChR current inhibition by AChR-Ab positive sera.

Nevertheless, we do not exclude other changes in AChR function when rapsyn is cotransfected into the cells. Rapsyn is a 43 kD membrane-associated cytoplasmic protein and has been shown to interact with the M3–M4 cytoplasmic loop of all AChR subunits.^21 22^ It is important to cluster and concentrate AChRs at the postsynaptic membrane of the neuromuscular junction, but rapsyn was also shown to modify AChR function by facilitation of recovery from desensitisation.^11^ Allosteric AChR modulation by rapsyn or by receptor–receptor interactions, when tightly clustered on the cell surface, could also have led to our observation that clustered AChR currents were more likely to be inhibited by AChR-Ab positive MG sera.

The MG samples used in our study represent the largest cohort used in an electrophysiological study so far, which was important to analyse systematically their effect on AChR function. We applied a very fast perfusion system and were thus able to resolve the time constants of desensitisation and recovery from desensitisation. Disease duration and therapeutic status of the cohort were heterogeneous, which might have biassed the correlation between inhibition of AChR function by MG sera with the clinical status. Samples from untreated, severely affected patients with MG with high antibody titres should ideally be used in future electrophysiological studies on AChR function to understand better the biological significance of current inhibition by MG sera, but this is difficult to accomplish as although MG is rare, it is now widely recognised and immunotherapies are often commenced early in the disease course, often before the patients attend a specialist centre.

The relevance of these findings to the in vivo situation in patients is difficult to assess. It is well established that AChR-Abs, that are divalent, induce both AChR internalisation through cross-linking adjacent receptors and also complement-dependent damage to the postsynaptic membrane. They are also known to inhibit binding of BTX to the AChR, which to some extent equates with the inhibition of AChR function that we have shown here,^23^ but most inhibitory studies have been performed on mouse cells and this is the first to look comprehensively at effects on human AChRs under conditions, RT and complement-inactivated sera, where neither internalisation or complement activation is likely to be relevant.

MG antibodies are highly heterogeneous^24 25^ and in vivo it is likely that all three mechanisms can be involved, depending on the specificities and affinities of the antibodies. The use of cells expressing clustered AChRs is an important development for the further characterisation of MG antibodies, and here provides novel evidence that puts the well-known blocking mechanism of AChR-Abs in a new perspective.
